# Genetic and molecular advancements in combined hepatocellular-cholangiocarcinoma: Addressing a persistent clinical conundrum

**DOI:** 10.17179/excli2026-9655

**Published:** 2026-08-20

**Authors:** Rabab Fatima

**Affiliations:** 1 School of Health Sciences & Technology, UPES, Dehradun 248007, India

## ⁯⁯⁯

Owing to the heterogeneous histomorphology and variable proportion presentation of cholangiocarcinoma (CCa) and hepatocellular carcinoma (HCC), accurate diagnosis and treatment of combined or mixed hepatocellular cholangiocarcinoma (CHC) remains a critical conundrum (Chu et al., 2024[2]). The rare hepatic neoplasm accounts for < 5 % of all primary hepatic neoplasms with insufficiently complex pre-operative diagnostic data. Liver-directed approaches such as hepatic arterial infusion chemotherapy, radioembolization, transarterial chemoembolization and ablative therapies fail to achieve adequate efficacy due to the overlapping biological and clinical patterns between its cancerous ingredients. The accuracy of alpha fetoprotein (AFP), CA 19-9 biomarkers as well as cross-sectional imaging for pre-operative diagnosis is hindered by the biphenotypic nature of the disease. Gemcitabine and cisplatin constitute the current first line treatment; survival remains dismal, highlighting the urgent need for comprehensive genetic and molecular profiling (Schizas et al., 2020[10]). Despite significant progress, the biphenotypic tumor remains incompletely characterized with a poor prognosis for diagnostically equivocal cases.

As per its nomenclature, CHC exhibits both HCC and CCa attributes; tissue samples from either of these areas, therefore, result in its misdiagnosis. Multiple biopsies from various tumor regions might prevent misclassification, but the uncertainty in considering the number of tumor regions for tumor type determination persists. In this context, a dual approach featuring a succinct combination of CT/MRI imaging and tumor biopsy improved the diagnostic outcome. Herein, 21 CHC samples resected between December 2006 and April 2017 were assessed retrospectively. Type 3 pattern derived from mixed imaging demonstrated moderate sensitivity (48 %), whereas biopsy alone showed limited sensitivity. The highest diagnostic accuracy was achieved via sequential combination of imaging followed by targeted biopsy with increased sensitivity (60 %) and appropriate specificity (82 %) (Gigante et al., 2019[3]). Comprehensive molecular characterization of CHC by exome sequencing (Es), DNA copy number detection, and gene expression profiling, revealed cholangiolocellular as a distinct biliary-derived entity with active TGF-β signalling and trivial chromosomal instability (Moeini et al., 2017[6]). An integrative genomic study involving Pan Asia multi-center (8 hospitals) with 133 CHC cases was reported. RNA sequencing, single nucleus sequencing (SNs), whole Es and whole genome sequencing (WGs), revealed CHC as a distinct subtype. By combining laser microdissection, SNs, and cancer cell fraction analysis, both monoclonal and multiclonal origins in individual type CHCs were identified, with monoclonal origins reported in all combined cases. Nestin expression was noticeably higher in CHCs, suggesting its utility as a biomarker for precise diagnosis (Xue et al., 2019[12]).

To elucidate complex molecular relationships among its tumoral components, Cazals-Hatem et al. (2004[1]) investigated loss of heterozygosity (LoH) along with CTNNB1 and TP53 mutation screening in 9 CCa, 15 CHC and 3 separate collision tumors (HCC and CCa lesions). Elevated chromosomal instability in both CHC and CCa was observed and CTNNB1 was found in only collision tumors. Moreover, recurrent LoH at 3p and 14q chromosomes was reported in more than 50 % of the CCa and the CHC cases and in fewer than 10 % of HCC cases, indicative of genetic closeness for CCa than HCC (Cazals-Hatem et al., 2004[1]). To improve diagnosis, Joseph et al. (2019[4]) performed captured-based next-generation sequencing of 20 CHC and 10 CCa samples (from cirrhotic patients) and demonstrated that CHC's molecular profile was more closely resembling HCC rather than the CCa component (Joseph et al., 2019[4]). Nonetheless, many studies have closely revealed genomic alterations in TERT promoters, AXIN 1 and IDH1/2 falling in HCC alone or/and in CCa as well. The most frequent alteration in CHC was seen in 45.3-80 %, 23-70 %, 0-11.8 %, and 10 % of evaluated cases, respectively (Sasaki et al., 2017[9]).

RNA sequencing, WGs and WEs on 10 CCa and 10 CHC samples in China revealed nuanced delineation in both mutational and transcriptional landscapes of both tumoral components of CHC. TP53 and CTNNB1 exhibited significant alterations in CHC along with KCNN3 in CCa. Notably, mutations in ARID1A, IDH1 and PBRM1 were common in CCa, whereas RYR3 and FBN2 were linked to metastasis and cell migration in CHC. HCC datasets were comparatively assessed using The Cancer Genome Atlas (Liu et al., 2018[5]). The diagnostic utility of CEUS (contrast enhanced ultrasound) and CT/MRI LI-RADS (liver imaging reporting & data systems) in 54 CHC and 55 HCCs deciphered a higher frequency of APHE (arterial phase hyperenhancement, 81.5 %) on CEUS and early washout in CHC, whereas RIM APHE was more prevalent on CT MRI (25.9 %) along with delayed enhancement (35.2 %). As per LI-RADS categorization, the majority of such lesions were categorized as either LR-M or LR-5 on both CEUS as well as MRI/CT (Zhou et al., 2022[15]). 7 CHC, along with other subtypes when sequenced using plasma cell-free DNA (cfDNA) fragmentomic, showed excellent sensitivity and specificity for cancerous and non-cancerous states, rendering circulating cells as prognostic biomarkers in resource limited settings (Zhang et al., 2022[14]).

To leverage the immunomodulatory therapeutic framework, 96 CHC samples were profiled using gene expression and targeted next-generation sequencing via nCounter PanCancer IO 360 Panel. The study revealed a subgroup of the tumor featuring intratumor immune activity and unique gene signatures for improved clinical outcomes (Nguyen et al., 2022[7]). In a thorough genomic analysis of viral and non-viral associated CHC cases via cBioPortal, longer survival with viral-infected cases was observed. Non-viral cases showed substantial mutational enrichment suggesting poorer prognosis, furthering consistent genomic annotations and validations (Puttagunta et al., 2026[8]). To identify new therapeutic targets and molecular distinctions between the tumor and its two components, 3 microarray Gene Expression Omnibus datasets for 77 HCC, 32 CCa and 34 CHC biochips were assessed by Meta Omics. 7313 differentially expressed genes with marked upregulation of S100A11 in CCa alone were evaluated. The robust transcriptomic platform revealed distinct tumor signalling pathways (Notch and Wnt), responsible for active molecular mechanisms and a peculiar tumor microenvironment (Xue et al., 2015[13]).

CHC remains a diagnostic challenge due to its dual lineage, heterogenous morphology and overlapping radiological and clinical features. Microvascular invasion, postoperative recurrence as well as survival rates have been well predicted by combining machine learning-based and radiomics models (Xiao et al., 2024[11]). A succinct integration of multi-omics with artificial intelligence and deep learning platforms provides novel perspectives for its classification, improving diagnostic precision and tumor-specific staging systems. In addition, the advent of digital medicine techniques such as 3D printing, contemporary therapeutic simulations and 3D organoid cancer technology may present enhanced guidance for preoperative complexities. Clinically translating these advances will strengthen prognostication, therapeutic decision-making and patient outcomes, thereby mitigating the overall burden of this rare and poorly understood malignancy.

## Declaration

### Conflict of interest

The author declares no conflict of interest.

### Artificial Intelligence (AI) - assisted technology

The author declares that no AI tool was used in writing the manuscript.
